# Supplementary material for: Post-pandemic changes in population immunity have reduced the likelihood of emergence of zoonotic coronaviruses
Source: Nat Commun. 2026 Mar 24;17:2248. doi: 10.1038/s41467-026-69988-8 (PMC13013692; doi:10.1038/s41467-026-69988-8)
Supplement: Supplementary file 1 — Supplementary Information [file 41467_2026_69988_MOESM1_ESM.pdf]

Supplementary Materials for

Post-pandemic changes in population immunity reduce the likelihood of emergence of zoonotic coronaviruses

This file contains:

Contents

1. Supplementary Figures 2
   1.1. Supplementary Figure 1: 2
   1.2. Supplementary Figure 2: 3
   1.3. Supplementary Figure 3: 4
   1.4. Supplementary Figure 4: 5
   1.5. Supplementary Figure 5: 6
   1.6. Supplementary Figure 6: 7
   1.7. Supplementary Figure 7: 8
   1.8. Supplementary Figure 8: 9
   1.9. Supplementary Figure 9: 10
   1.10. Supplementary Figure 10: 11
   1.11. Supplementary Figure 11: 11
   1.12. Supplementary Figure 12: 12
   1.13. Supplementary Figure 13: 12
   1.14. Supplementary Figure 14: 13
2. Supplementary Tables 13
   2.1. Supplementary Table 1: 13
   2.2. Supplementary Table 2: 13
   2.3. Supplementary Table 3: 13
   2.4. Supplementary Table 4: 14
   2.5. Supplementary Table 5: 14
   2.6. Supplementary Table 6: 14
   2.7. Supplementary Table 7: 14
   2.8. Supplementary Table 8: 18
   2.9. Supplementary Table 9: 19
3. Supplementary References 19

1. Supplementary Figures

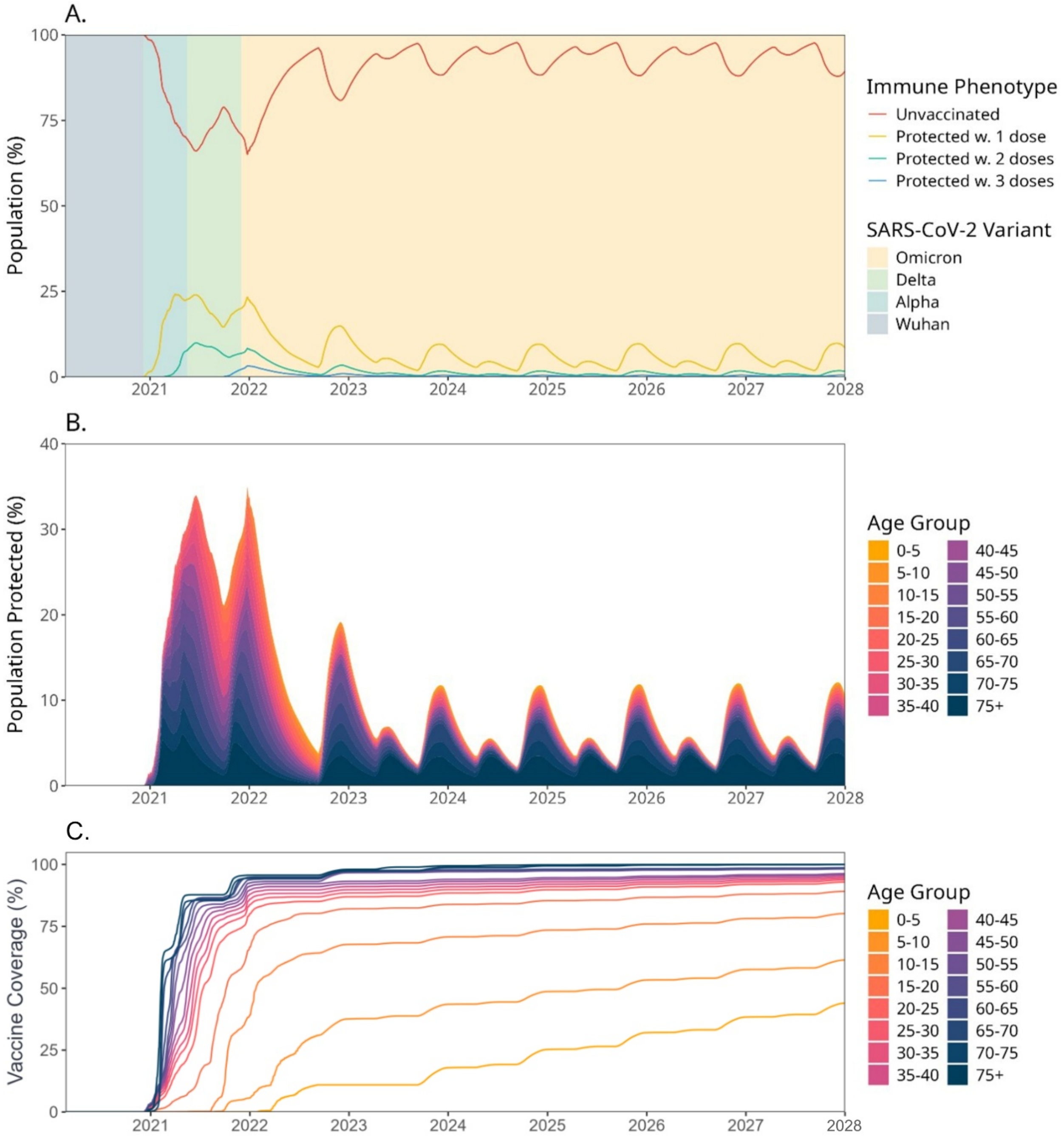

1.1. Supplementary Figure 1: Population coverage of vaccination protection over time. A) Lines show the number of individuals with an unvaccinated (red), protected with one dose (yellow), two doses (green), or three doses (blue) immune phenotype as a percentage of the total population over time, in an example model run under similar conditions to Figure 2C, but with an additional vaccination level and no preventative vaccine program (i.e., only real-world programs and seasonal vaccination). The predominant SARS-CoV-2 variant is indicated by the shaded background area for reference. B) The percentage of individuals in the population protected by vaccination over time in the three-level (unvaccinated, 1 dose, 2 dose) model used throughout this study, separated by age group. C) Lines show vaccine coverage (i.e., history, as opposed to a transient vaccine protection phenotype) over time, separated by age group.

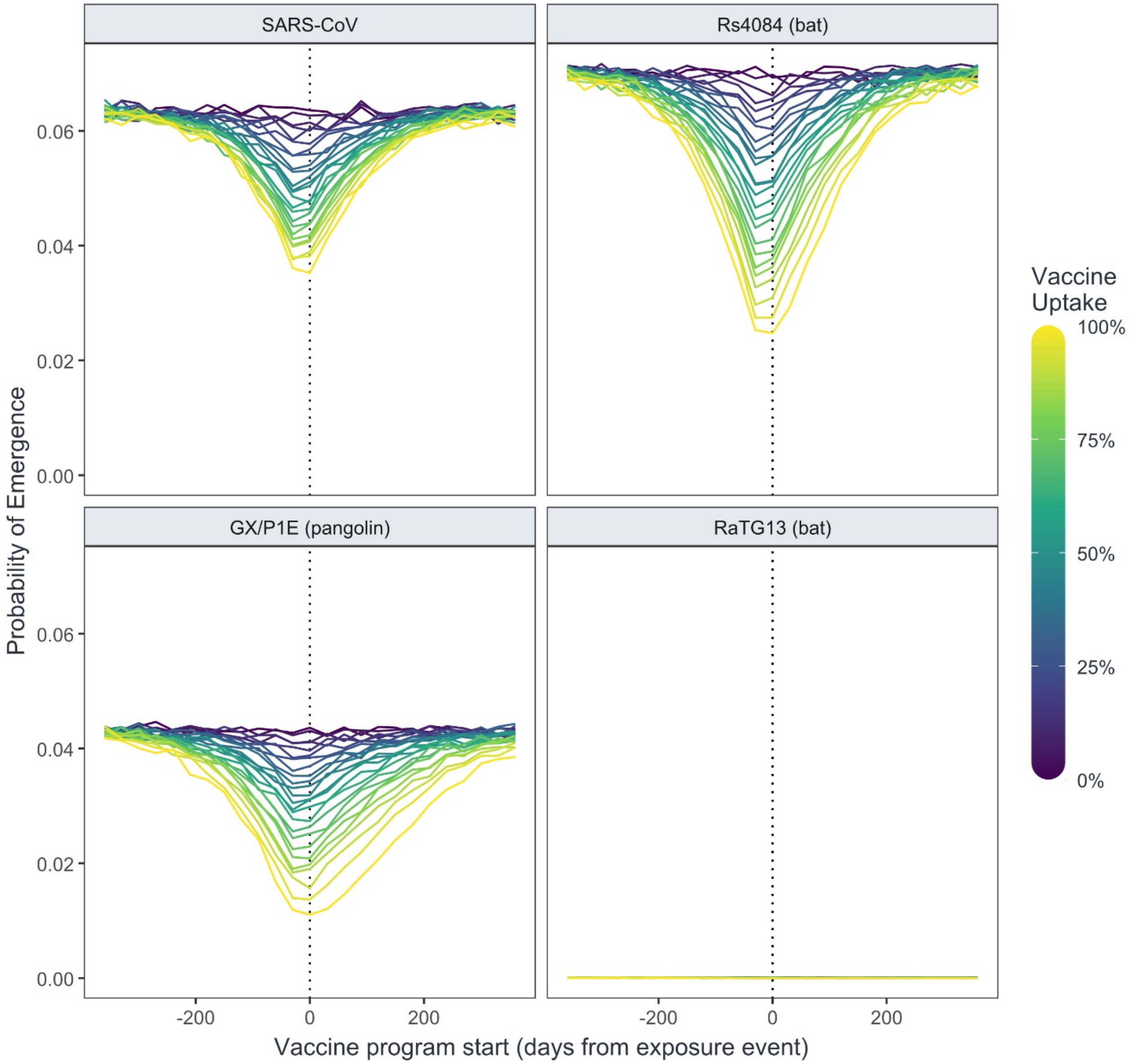

1.2. Supplementary Figure 2: Probability of emergence of different SARS coronaviruses in the presence of vaccination and co-circulating SARS-CoV-2. Lines represent the point estimates of the probability of emergence for four SARS coronaviruses in a population with co-circulating SARS-CoV-2 under preventative vaccination programs at different times relative to the SARS-CoV-X exposure event. The colour of each line indicates the (%) uptake of the preventative vaccination program.

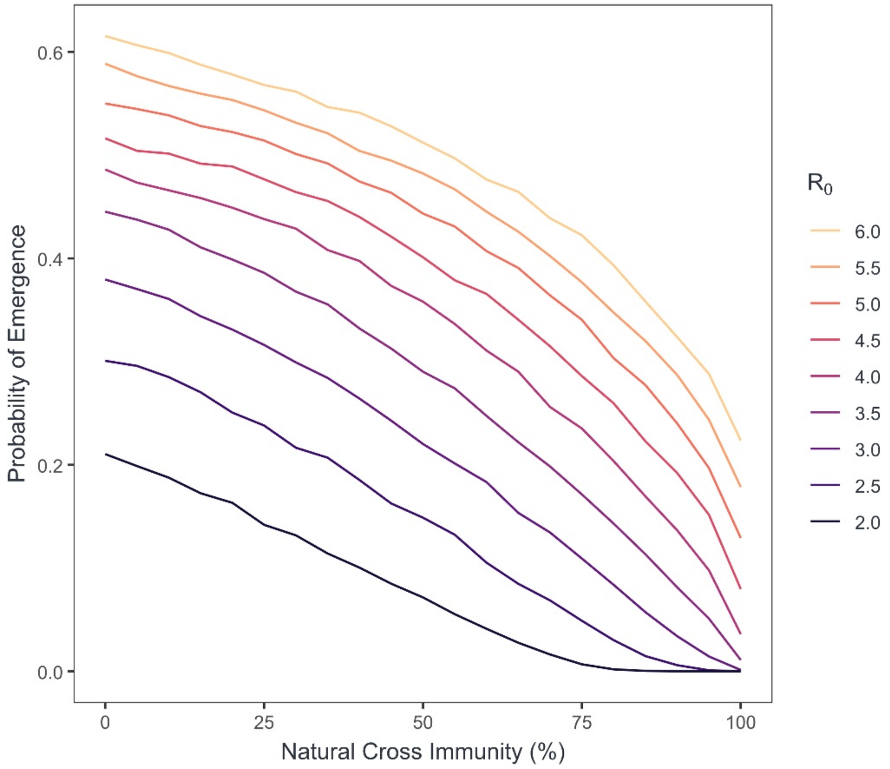

1.3. Supplementary Figure 3: Probability of emergence of theoretical SARS coronaviruses with different R_0 and levels of natural cross-immunity to SARS-CoV-2. Lines represent the point estimates of the probability of emergence of theoretical SARS coronaviruses with different R0 values (colour) and levels of natural cross-immunity to SARS-CoV-2.

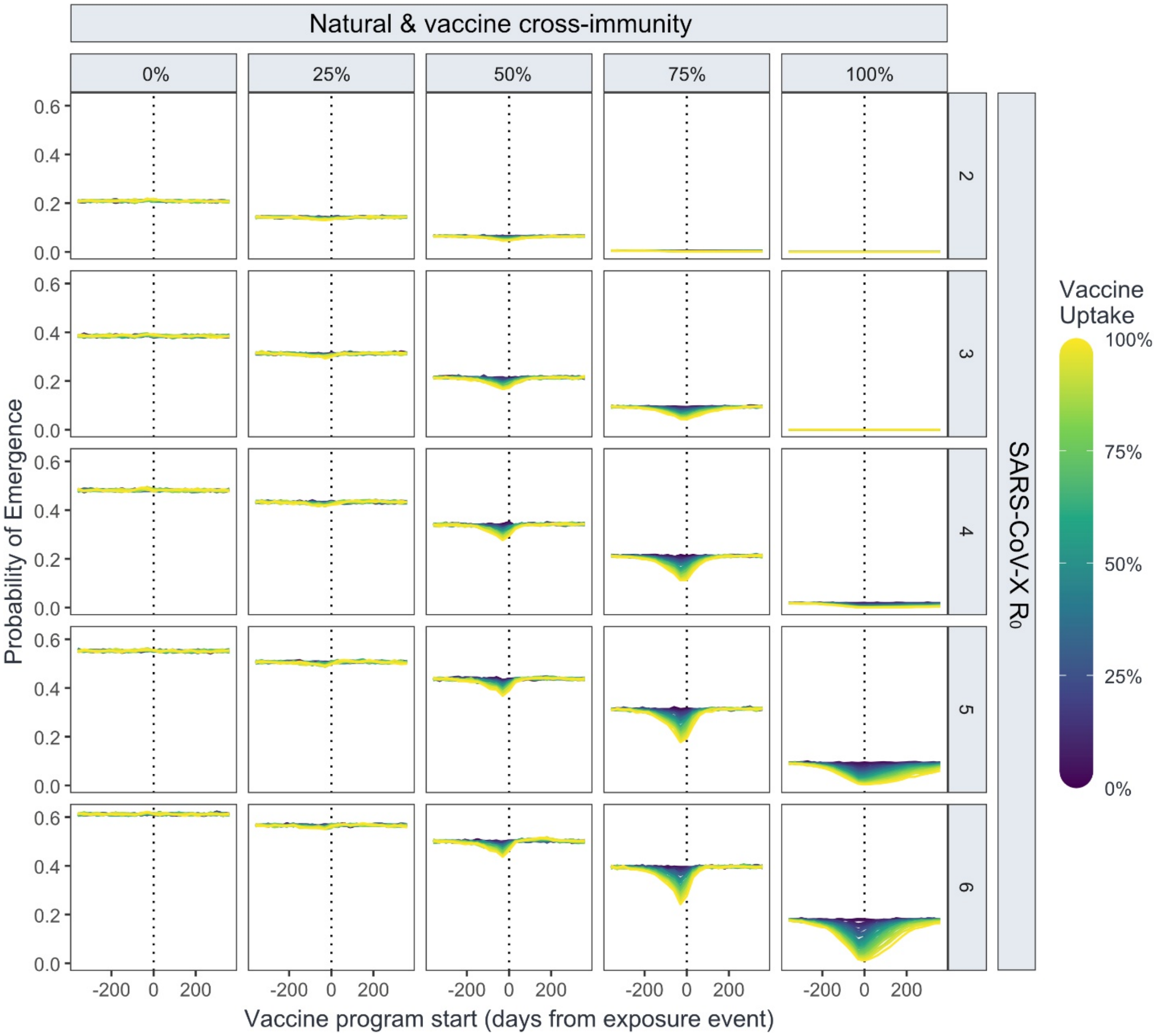

1.4. Supplementary Figure 4: Probability of emergence of theoretical SARS coronaviruses under conditions of equal cross-immunity and vaccine effectiveness. Lines show point estimates of the probability of emergence for 25 theoretical SARS coronaviruses with different R0 values (facet rows) and conditions of cross-immunity and vaccine effectiveness (facet columns) in a population with co-circulating SARS-CoV-2. In these scenarios, protection against SARS-CoV-X infection conferred from recovering from natural infection with SARS-CoV-2 ("natural cross-immunity") and vaccination ("vaccine cross-immunity") are identical.

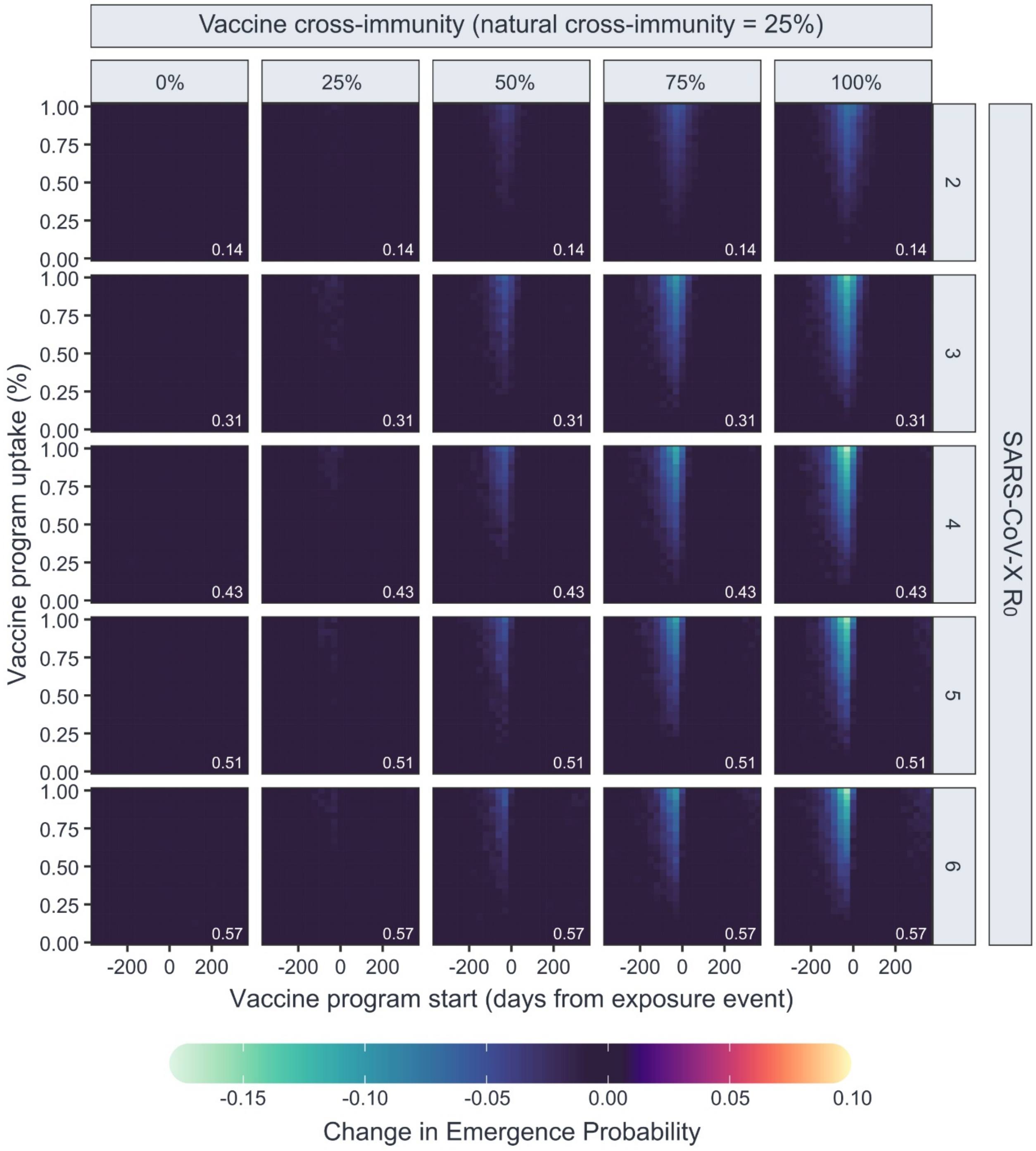

1.5. Supplementary Figure 5: Probability of emergence of theoretical SARS coronaviruses under conditions of varying vaccine cross-immunity. Heatmaps show point estimates of the probability of emergence for 25 theoretical SARS coronaviruses with different R0 values (facet rows) and varying conditions of vaccine cross-immunity (facet columns) in a population with co-circulating SARS-CoV-2. In these scenarios, natural cross-immunity has been fixed at 25%.

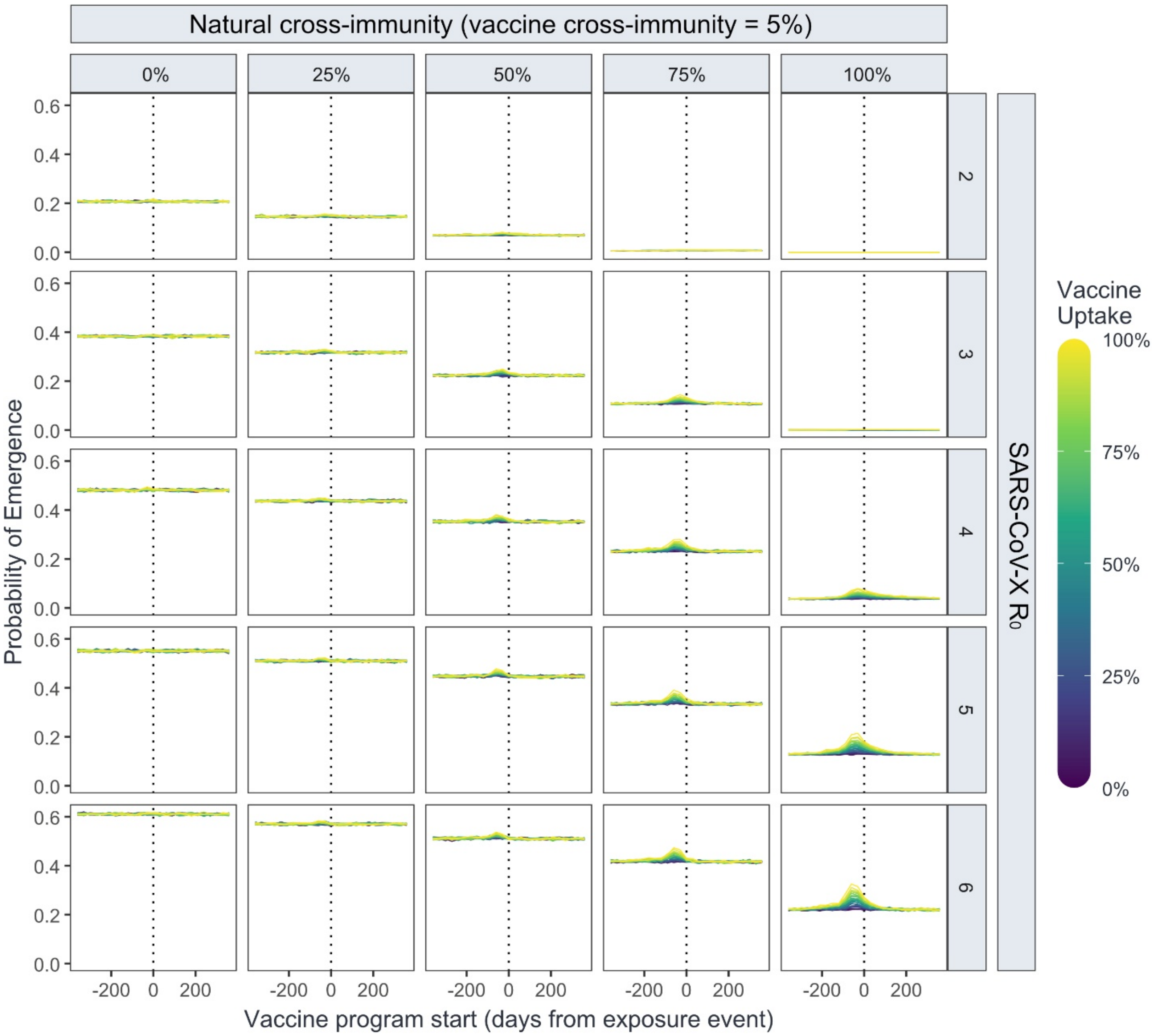

1.6. Supplementary Figure 6: Probability of emergence of theoretical SARS coronaviruses under conditions of low vaccine cross-immunity and high natural cross-immunity. Lines show point estimates of the probability of emergence for 25 theoretical SARS coronaviruses with different R0 values (facet rows) and varying conditions of natural cross-immunity (facet columns) in a population with co-circulating SARS-CoV-2 and low (5%) vaccine cross-immunity.

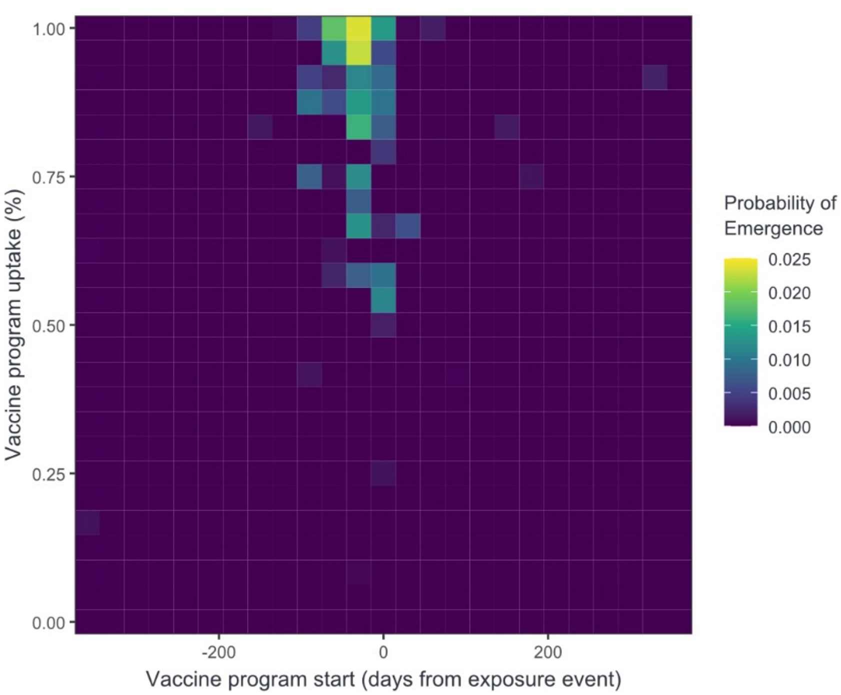

1.7. Supplementary Figure 7: Probability of emergence of theoretical SARS coronaviruses incapable of emergence without detrimental vaccination. Heatmap shows point estimates of the probability of emergence for a theoretical sarbecovirus of $R_0 = 2$, natural cross-immunity to SARS-CoV-2 of 80%, and under a preventative vaccine campaign using a 5% cross-reactive vaccine.

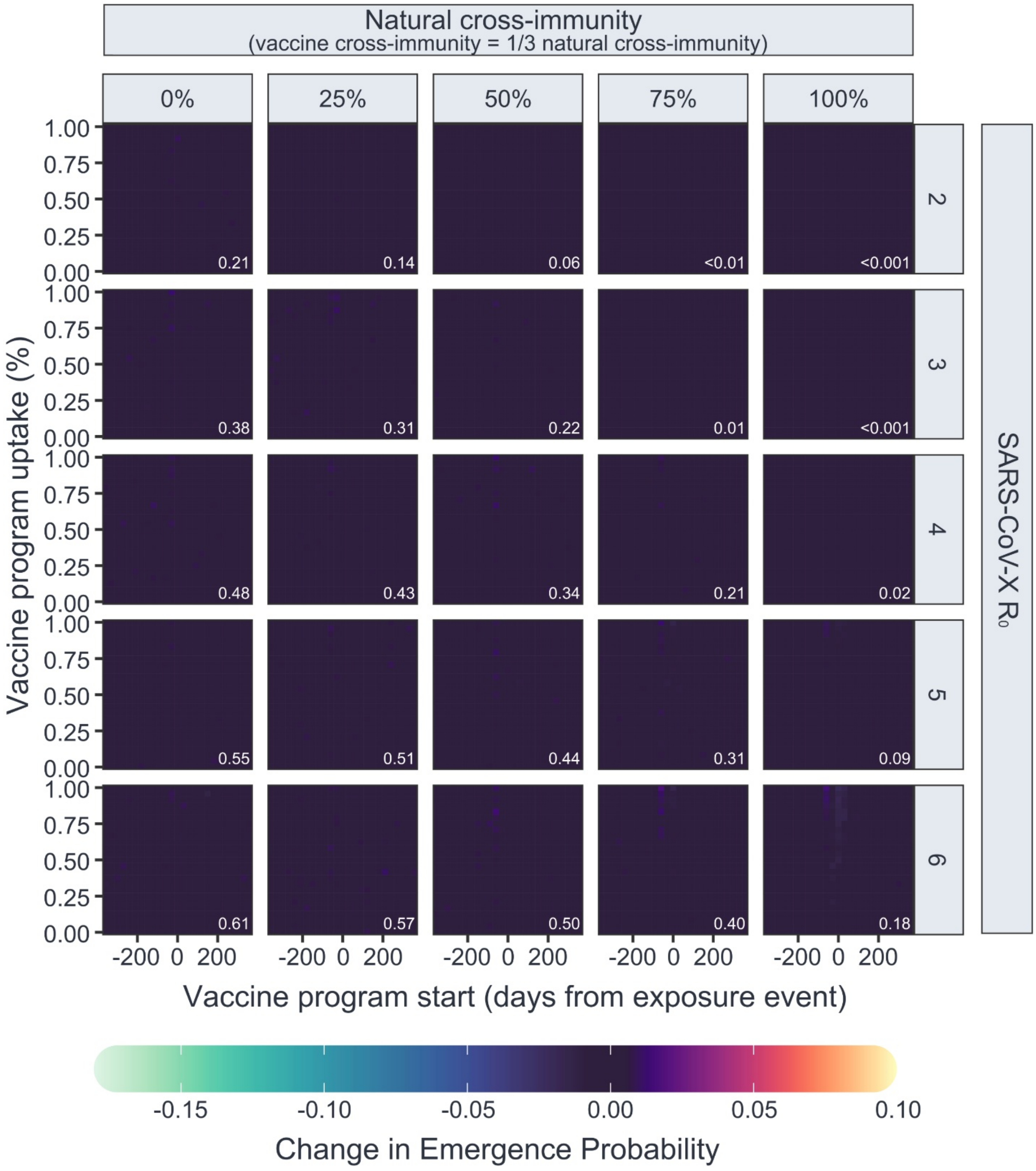

1.8. Supplementary Figure 8: Probability of emergence of theoretical SARS coronaviruses in conditions where vaccine cross-immunity is one-third that of natural cross-immunity. Heatmaps show point estimates of the probability of emergence for 25 theoretical SARS coronaviruses with different R0 values (facet rows) and varying conditions of natural cross-immunity (facet columns) in a population with co-circulating SARS-CoV-2. In these scenarios, vaccine cross-immunity has been set to one-third of the level of natural cross-immunity.

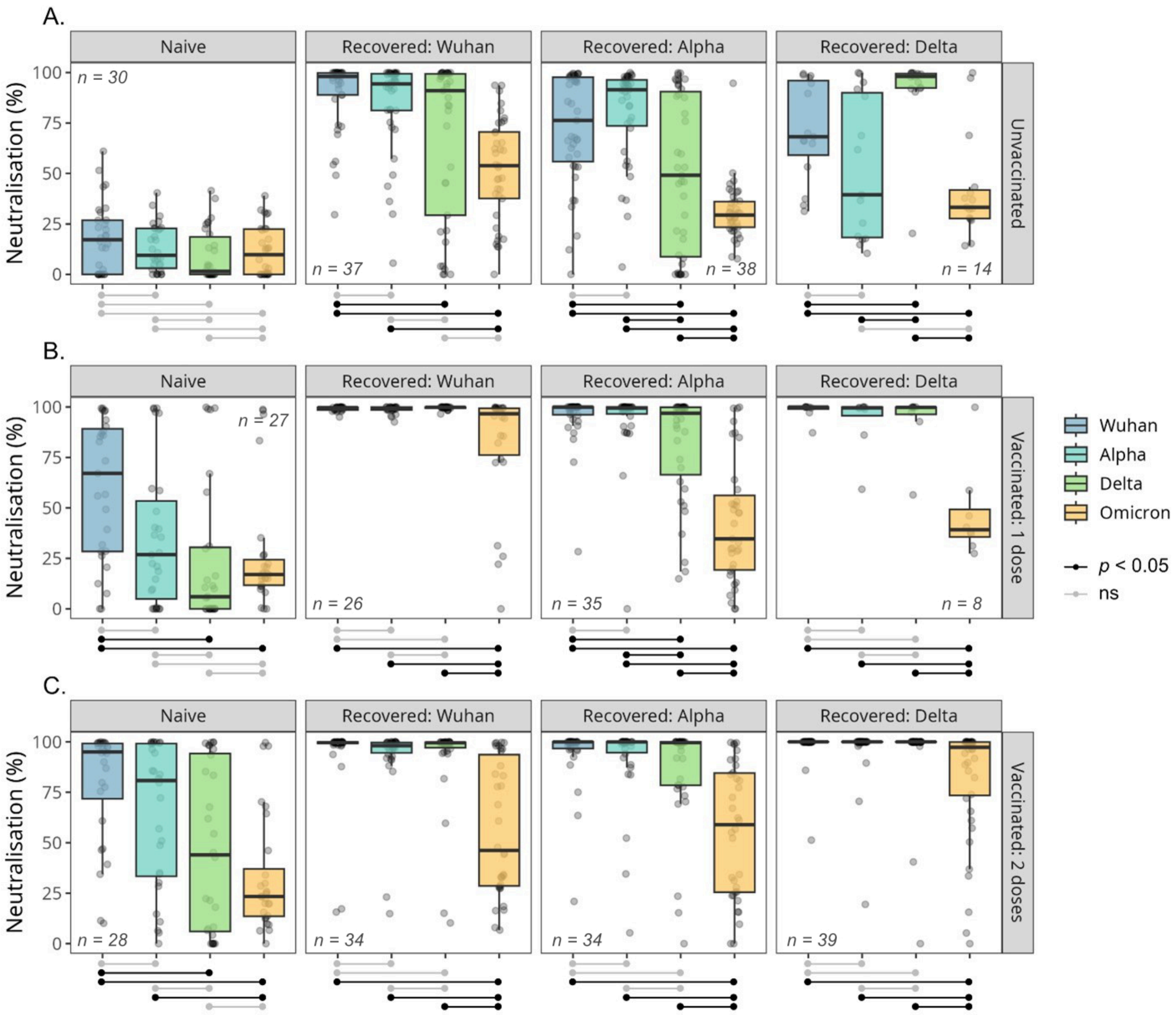

1.9. Supplementary Figure 9: Neutralisation of viral pseudotypes carrying the spike proteins of different SARS-CoV-2 variants by sera from individuals of different infection and vaccination histories. Boxplots show the percentage neutralisation of pseudotype viruses by sera from individuals who were unvaccinated (A), vaccinated once (B), or vaccinated twice (C) against SARS-CoV-2. Results are separated into subplots (columns) based on an individual's history of natural infection, and separate boxplots are shown for neutralisation of pseudoviruses with Wuhan (blue), Alpha (cyan), Delta (green) and Omicron (yellow) spike proteins. Significant differences in the strength of neutralisation are shown with black horizontal lines below each subplot, assessed using Welch's t-tests with Holm correction for multiple testing.

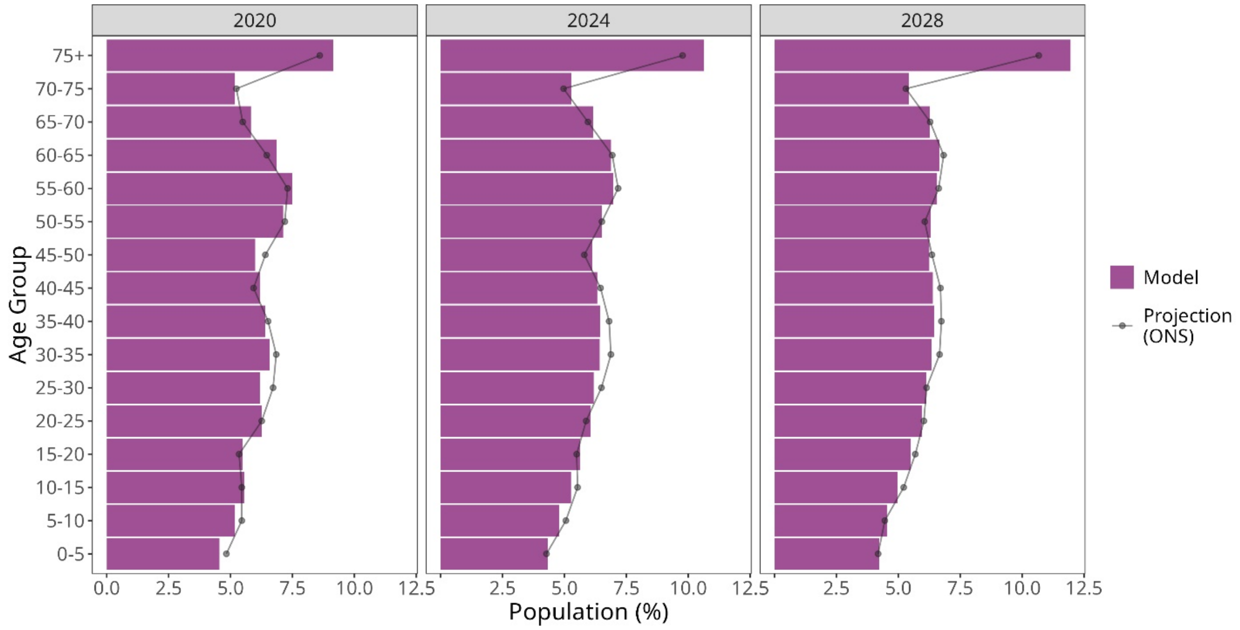

1.10. Supplementary Figure 10: Population percentages by age-group over time. Orange bars show the number of individuals in each age group as a percentage of the total population on January 1st 2020, 2024, and 2028. Grey lines indicate the population projections for Scotland estimated by the Office for National Statistics (ONS), available from: https://www.nrscotland.gov.uk/statistics-and-data/population-migration-and-households/

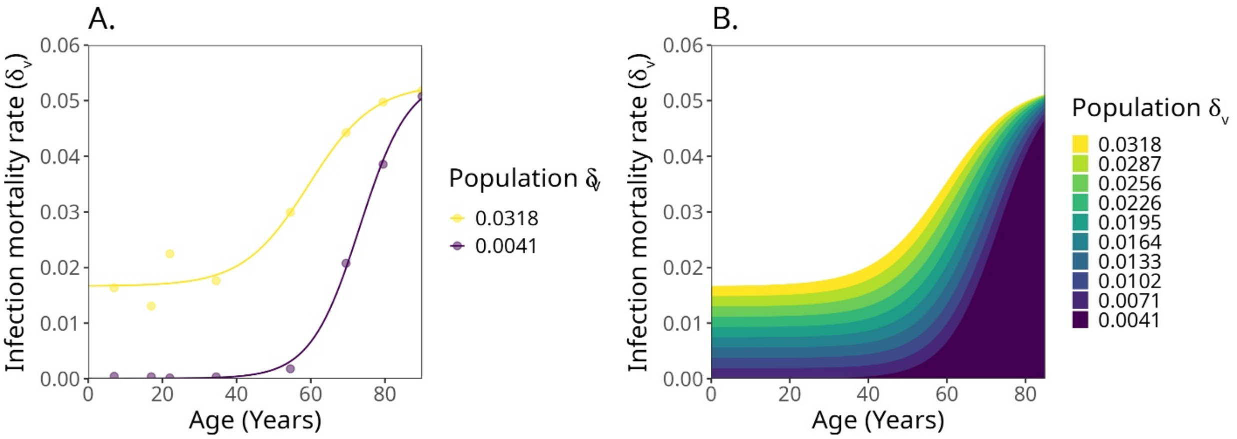

1.11. Supplementary Figure 11: Inferring infection mortality rate across age-groups for theoretical SARS coronaviruses of different population infection mortality rates. A) Infection mortality rates of different ages for SARS-CoV-2 (purple) and MERS-CoV (yellow) with a four-parameter logistic non-linear least squares model fitted to each group. B) Using the model fit from (A), infection mortality rates of different ages are inferred for viruses with different population infection mortality rates.

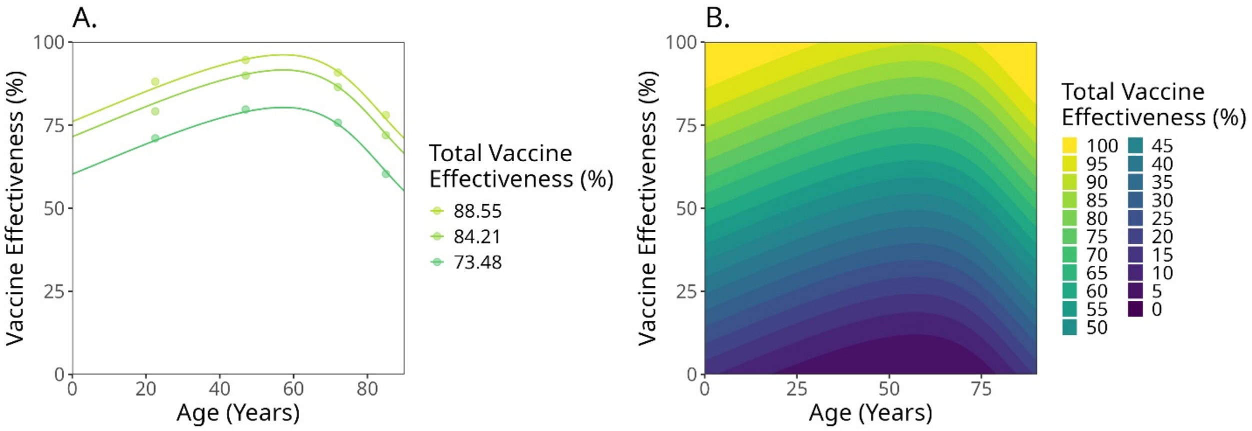

1.12. Supplementary Figure 12: Inferring the levels of immune protection across age groups for different levels of population protection. A) Estimates of COVID-19 vaccine effectiveness against re-infection for different ages for those who received a 2nd dose within 3 months (light green), 3-6 months (mid green), or later than 6 months (dark green) from their 1st dose. A double-logistic non-linear least squares model is fitted to each group. B) Using the model fit from (A), the levels of immune protection across different ages are inferred for sources of immunity with different levels of population protection.

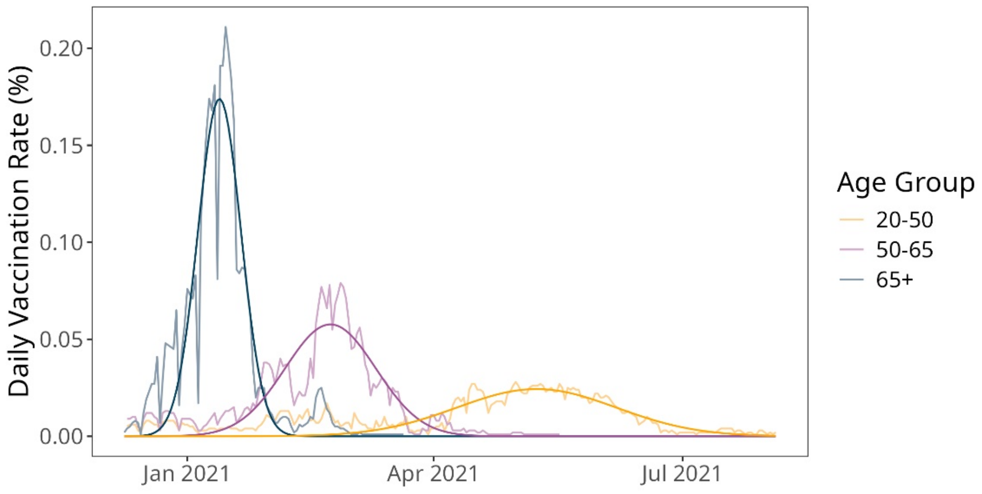

1.13. Supplementary Figure 13: The rate of administration of the first COVID-19 vaccine dose in Scotland. Data on the daily rate of vaccination for the first COVID-19 dose (faded lines) is shown for age groups 65+ (dark blue), 50-65 (purple), and 20-50 (yellow). Non-normalised Gaussian curves, fitted using a non-linear least squares approach, are overlaid in darker lines.

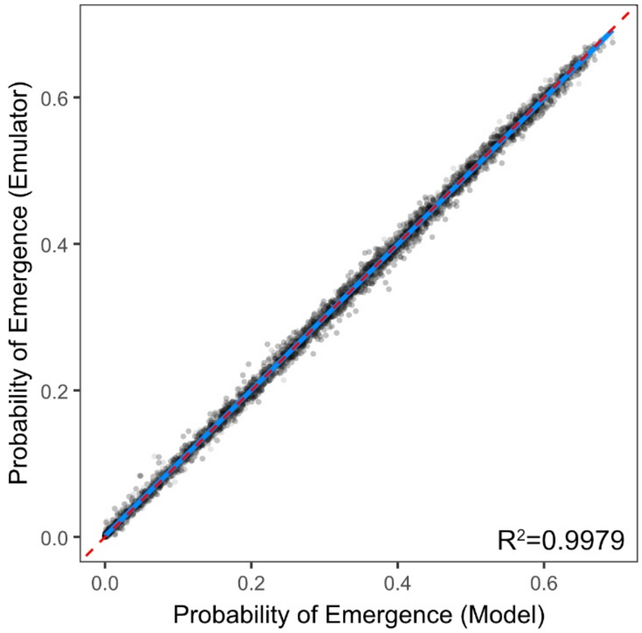

1.14. Supplementary Figure 14: GBRT emulator model evaluation. Estimated probability of SARS-CoV-X emergence across a 5,000 Latin Hypercube parameter sampling scheme of 9D parameter space taken directly from the epidemiological model (x-axis) and predicted from the GBRT emulator model (y-axis). The trendline for a least-squares linear model (y ~ βx +c) is shown in blue, and a theoretical 1:1 trendline as a dashed red line.

2. Supplementary Tables

2.1. Supplementary Table 1: Across-viruses mixed effect model random effects.

Group	Variance	Std. Deviation
Serum.ID	296.3	17.21
Virus	451.2	21.24
Residual	358.7	18.94

2.2. Supplementary Table 2: Across-viruses mixed effect model fixed effects. Intercept = Naïve & Unvaccinated

Effect	Estimate (μ)	Std. Error	df	t-value	p-value
(Intercept)	14.820	11.211	3.706	1.322	0.2619
Recovered	26.877	4.148	344	6.480	$3.2x10^{-10}$
Vaccinated 1 dose	30.347	5.212	344	5.823	$1.3x10^{-8}$
Vaccinated 2 doses	39.677	5.162	344	7.686	$1.6x10^{-13}$
Recovered : 1 dose	-7.918	6.090	344	-1.300	0.1944
Recovered : 2 doses	-14.348	5.882	344	-2.439	0.0152

2.3. Supplementary Table 3: Across-immune group mixed effect model random effects.

Group	Variance	Std. Deviation
Serum.ID	296.45	17.218
Recovered	181.55	13.474
Vaccinated	265.05	16.280
Recovered : Vaccinated	15.72	3.965
Residual	358.65	18.938

2.4. Supplementary Table 4: Across-immune group mixed effect model fixed effects. Intercept = SARS-CoV-1

Effect	Estimate (μ)	Std. Error	df	t-value	p-value
(Intercept)	29.966	13.565	2.553	2.209	0.13
Rs4084	9.761	1.432	1047	6.818	$1.6x10^{-11}$
GX/P1E	13.349	1.432	1047	9.325	$6.4x10^{-20}$
RaTG13	48.713	1.432	1047	34.028	$1.6x10^{-171}$

2.5. Supplementary Table 5: Across-immune group mixed effect model random effects.

Group	Variance	Std. Deviation
Serum.ID	267.39	16.352
Recovered	52.44	5.914
Vaccinated	163.26	7.241
Recovered : Vaccinated	34.98	12.777
Residual	433.28	20.815

2.6. Supplementary Table 6: Across-immune group mixed effect model fixed effects.

Effect	Estimate (μ)	Std. Error	df	t-value	p-value
(Intercept)	37.49292	8.52023	2.58108	4.40	0.0295
Similarity^2	0.09249	0.00299	958.99953	30.93	$3.0x10^{-146}$

2.7. Supplementary Table 7: Parameterization Values and References. Values are given as quantities (population sizes), daily rates (migration) or daily per-capita rates (all other values).

Parameter	Population/Virus	Value(s)		Reference(s)
Population Size	Scotland (by age)	0-4:	247737	[1]
		5-9:	281912	
		10-14:	303266	
		15-19:	298467	
		20-24:	341371	
		25-29:	337679	
		30-34:	357840	
		35-39:	348532	
		40-44:	336578	
		45-49:	326504	
		50-54:	388254	
		55-59:	408491	
		60-64:	373674	
		65-69:	317469	
		70-74:	282016	
		75+:	497910	
Birth Rate	Scotland (by age)	0-4:	0	[2]
		5-9:	0	
		10-14:	0.0000002444	
		15-19:	0.0000166204	
		20-24:	0.0000478186	
		25-29:	0.0000952367	
		30-34:	0.0001204575	
		35-39:	0.0000601418	
		40-44:	0.0000110273	
		45-49:	0.0000005861	
		50-54:	0.0000000335	

		55-59: 0<br>60-64: 0<br>65-69: 0<br>70-74: 0<br>75+: 0	
Crude Death Rate	Scotland (by age)	0-4: 0.00000187412<br>5-9: 0.00000023801<br>10-14: 0.00000047469<br>15-19: 0.00000111622<br>20-24: 0.00000154121<br>25-29: 0.00000211211<br>30-34: 0.00000305548<br>35-39: 0.00000481195<br>40-44: 0.00000682835<br>45-49: 0.00000915344<br>50-54: 0.00001264592<br>55-59: 0.00001855913<br>60-64: 0.00002881181<br>65-69: 0.00004558282<br>70-74: 0.00007269524<br>75+: 0.00018167223	[2]
Net Migration Rate	Scotland (by age)	0-4: 3<br>5-9: 3<br>10-14: 2<br>15-19: 14<br>20-24: 13<br>25-29: 2<br>30-34: 5<br>35-39: 3<br>40-44: 2<br>45-49: 2<br>50-54: 2<br>55-59: 2<br>60-64: 2<br>65-69: 1<br>70-74: 0<br>75+: 0	[1]
Contact Rates	Scotland (by age)	Multi-dimensional. Available at: https://github.com/ryanmimrie/Publications_2025_SARS-CoV-X-Emergence	[3–5]
Vaccination Rates	Scotland (by age)	Multi-dimensional. Available at: https://github.com/ryanmimrie/Publications_2025_SARS-CoV-X-Emergence	[6,7]
Vaccination Waning Rate	Scotland	0.01131	Inferred through exponential decay model fitted to data in [8]
SARS-CoV-2 Variant Prevalences	Scotland	Multi-dimensional. Available at: https://github.com/ryanmimrie/Publications_2025_SARS-CoV-X-Emergence	[9]
Incubation Rate	SARS-CoV-2 (Wuhan)	0.217	[10]

	SARS-CoV-2 (Alpha)	0.202	[10]
	SARS-CoV-2 (Delta)	0.226	[10]
	SARS-CoV-2 (Omicron)	0.277	[10]
	SARS-CoV-1	0.217	Assumed equal to SARS-CoV-2 (Wuhan)
	Rs4084	0.217	Assumed equal to SARS-CoV-2 (Wuhan)
	GX/P1E	0.217	Assumed equal to SARS-CoV-2 (Wuhan)
	RaTG13	0.217	Assumed equal to SARS-CoV-2 (Wuhan)
Infection Mortality Rate	SARS-CoV-2 (Wuhan) (by age)	0-5: 0.00000364<br>5-10: 0.0000059<br>10-15: 0.00000956<br>15-20: 0.00001575<br>20-25: 0.00002552<br>25-30: 0.00004133<br>30-35: 0.00006703<br>35-40: 0.00010903<br>40-45: 0.000178<br>45-50: 0.00028885<br>50-55: 0.00046478<br>55-60: 0.00074241<br>60-65: 0.00118192<br>65-70: 0.00187175<br>70-75: 0.00285011<br>75+: 0.0058782	[11]
	SARS-CoV-2 (Alpha) (by age)	0-5: 0.00000364<br>5-10: 0.0000059<br>10-15: 0.00000956<br>15-20: 0.00001575<br>20-25: 0.00002552<br>25-30: 0.00004133<br>30-35: 0.00006703<br>35-40: 0.00010903<br>40-45: 0.000178<br>45-50: 0.00028885<br>50-55: 0.00046478<br>55-60: 0.00074241<br>60-65: 0.00118192<br>65-70: 0.00187175<br>70-75: 0.00285011<br>75+: 0.0058782	[11]
	SARS-CoV-2 (Delta) (by age)	0-5: 0.00000364<br>5-10: 0.0000059<br>10-15: 0.00000956<br>15-20: 0.00001575<br>20-25: 0.00002552<br>25-30: 0.00004133<br>30-35: 0.00006703<br>35-40: 0.00010903<br>40-45: 0.000178<br>45-50: 0.00028885<br>50-55: 0.00046478<br>55-60: 0.00074241<br>60-65: 0.00118192	[11]

	65-70:	0.00187175	
	70-75:	0.00285011	
	75+:	0.0058782	
SARS-CoV-2 (Omicron) (by age)	0-5:	0.00000364	[11]
	5-10:	0.0000059	
	10-15:	0.00000956	
	15-20:	0.00001575	
	20-25:	0.00002552	
	25-30:	0.00004133	
	30-35:	0.00006703	
	35-40:	0.00010903	
	40-45:	0.000178	
	45-50:	0.00028885	
	50-55:	0.00046478	
	55-60:	0.00074241	
	60-65:	0.00118192	
	65-70:	0.00187175	
	70-75:	0.00285011	
	75+:	0.0058782	
SARS-CoV-1 (by age)	0-5:	0.00006223	Inferred from non-linear least squares model (Supplementary Figure 11) with population estimate taken from [12]
	5-10:	0.00010077	
	10-15:	0.00016279	
	15-20:	0.00026709	
	20-25:	0.00042973	
	25-30:	0.00068816	
	30-35:	0.00109603	
	35-40:	0.00173207	
	40-45:	0.00270132	
	45-50:	0.00409076	
	50-55:	0.00595211	
	55-60:	0.0082628	
	60-65:	0.01090161	
	65-70:	0.01361388	
	70-75:	0.0159554	
	75+:	0.01908244	
Rs4084 (by age)	0-5:	0.00006223	Assumed equal to SARS-CoV-1
	5-10:	0.00010077	
	10-15:	0.00016279	
	15-20:	0.00026709	
	20-25:	0.00042973	
	25-30:	0.00068816	
	30-35:	0.00109603	
	35-40:	0.00173207	
	40-45:	0.00270132	
	45-50:	0.00409076	
	50-55:	0.00595211	
	55-60:	0.0082628	
	60-65:	0.01090161	
	65-70:	0.01361388	
	70-75:	0.0159554	
	75+:	0.01908244	
GX/P1E (by age)	0-5:	0.00000364	Assumed equal to SARS-CoV-2 (Wuhan)
	5-10:	0.0000059	
	10-15:	0.00000956	
	15-20:	0.00001575	
	20-25:	0.00002552	

		25-30: 0.00004133	
		30-35: 0.00006703	
		35-40: 0.00010903	
		40-45: 0.000178	
		45-50: 0.00028885	
		50-55: 0.00046478	
		55-60: 0.00074241	
		60-65: 0.00118192	
		65-70: 0.00187175	
		70-75: 0.00285011	
		75+: 0.0058782	
	RaTG13 (by age)	0-5: 0.00000364	Assumed equal to SARS-CoV-2 (Wuhan)
		5-10: 0.0000059	
		10-15: 0.00000956	
		15-20: 0.00001575	
		20-25: 0.00002552	
		25-30: 0.00004133	
		30-35: 0.00006703	
		35-40: 0.00010903	
		40-45: 0.000178	
		45-50: 0.00028885	
		50-55: 0.00046478	
		55-60: 0.00074241	
		60-65: 0.00118192	
		65-70: 0.00187175	
		70-75: 0.00285011	
		75+: 0.0058782	

2.8. Supplementary Table 8: Prior and approximate posterior distributions for fitted parameters of SARS-CoV-2 variants. Parameters were assigned lognormal priors with the indicated mean (μ) and standard deviation (σ) on the log scale. For each parameter, the table reports the mean and 95% HPD interval of the prior distribution (on the original scale) and the approximate posterior distribution obtained from ABC fitting.

SARS-CoV-2 strain	Parameter	Prior (Lognormal)	Prior Summary: Mean (95% CIs)	Posterior Summary (Mean, 95% CI)
Wuhan	R_0	μ = 1, σ = 0.5	3.08 (1.02, 7.24)	1.58 (1.55, 1.61)
	Recovery rate	μ = -1, σ = 0.5	0.42 (0.13, 0.98)	0.050 (0.047, 0.052)
	Waning rate	μ = -5, σ = 2	0.04 (0.00, 0.34)	0.006 (0.000, 0.025)
Alpha	R_0	μ = 1, σ = 0.5	3.08 (1.02, 7.24)	2.29 (2.11, 2.44)
	Recovery rate	μ = -1, σ = 0.5	0.42 (0.13, 0.98)	0.387 (0.300, 0.447)
	Waning rate	μ = -5, σ = 2	0.04 (0.00, 0.34)	0.001 (0.000, 0.002)
Delta	R_0	μ = 1, σ = 0.5	3.08 (1.02, 7.24)	2.58 (2.37, 2.83)
	Recovery rate	μ = -1, σ = 0.5	0.42 (0.13, 0.98)	0.089 (0.058, 0.118)
	Waning rate	μ = -5, σ = 2	0.04 (0.00, 0.34)	0.002 (0.000, 0.007)
Omicron	R_0	μ = 1, σ = 0.5	3.08 (1.02, 7.24)	3.31 (2.70, 4.04)
	Recovery rate	μ = -1, σ = 0.5	0.42 (0.13, 0.98)	0.373 (0.298, 0.488)
	Waning rate	μ = -5, σ = 2	0.04 (0.00, 0.34)	0.004 (0.000, 0.010)

2.9. Supplementary Table 9: Emergence probability estimates for the sarbecovirus panel in current and naïve populations.

Virus	Population Immunity	Probability of Emergence (95% CIs)
SARS-CoV	Naïve	0.206 (0.181, 0.232)
Rs4084	Naïve	0.201 (0.177, 0.227)
GX/P1E	Naïve	0.203 (0.178, 0.229)
RaTG13	Naïve	0.202 (0.178, 0.228)
SARS-CoV	Current Conditions	0.0702 (0.0699, 0.0705)
Rs4084	Current Conditions	0.0632 (0.0629, 0.0635)
GX/P1E	Current Conditions	0.0432 (0.0430, 0.0435)
RaTG13	Current Conditions	0.0025 (0.0019, 0.0033)

3. Supplementary References

1. NRS. Population, migration and households - National Records of Scotland (NRS). (accessed 04/02/2026) https://www.nrscotland.gov.uk/statistics-and-data/population-migration-and-households/#.

2. NRS. Births, deaths, marriages and life expectancy - National Records of Scotland (NRS). (accessed 04/02/2026) https://www.nrscotland.gov.uk/statistics-and-data/births-deaths-marriages-and-life-expectancy/#.

3. Mossong, J. *et al.* Social Contacts and Mixing Patterns Relevant to the Spread of Infectious Diseases. *PLoS Med.* **5**, e74 (2008).

4. Gimma, A. *et al.* Changes in social contacts in England during the COVID-19 pandemic between March 2020 and March 2021 as measured by the CoMix survey: A repeated cross-sectional study. *PLoS Med.* **19**, e1003907 (2022).

5. Jarvis, C. I. *et al.* Social contact patterns following the COVID-19 pandemic: a snapshot of post-pandemic behaviour from the CoMix study. *Epidemics* **48**, 100778 (2024).

6. PHS. COVID-19 Vaccination in Scotland up to September 2022 - Daily Trend of Vaccinations by Age Group and Sex (accessed 04/02/2026) https://www.opendata.nhs.scot/dataset/covid-19-vaccination-in-scotland/resource/9b99e278-b8d8-47df-8d7a-a8cf98519ac1.

7. PHS. Vaccination Surveillance. (accessed 04/02/2026) https://scotland.shinyapps.io/phs-vaccination-surveillance/.

8. Arunachalam, P. S. *et al.* Durability of immune responses to the booster mRNA vaccination against COVID-19. *J. Clin. Investig.* **133**, e167955 (2023).

9. Whitaker, M. *et al.* Variant-specific symptoms of COVID-19 in a study of 1,542,510 adults in England. *Nat. Commun.* **13**, 6856 (2022).

10. Galmiche, S. *et al.* SARS-CoV-2 incubation period across variants of concern, individual factors, and circumstances of infection in France: a case series analysis from the ComCor study. *Lancet Microbe* **4**, e409–e417 (2023).

11. Team, C.-19 F. Variation in the COVID-19 infection–fatality ratio by age, time, and geography during the pre-vaccine era: a systematic analysis. *Lancet* **399**, 1469–1488 (2022).

12. WHO. Consensus document on the epidemiology of severe acute respiratory syndrome (SARS). (accessed 04/02/2026)

https://iris.who.int/bitstream/handle/10665/70863/WHO_CDS_CSR_GAR_2003.11_eng.pdf?sequence=1&isAllowed=y.
